# Microstructure, Mechanical Properties, and Corrosion Resistance of Thermomechanically Processed AlZn6Mg0.8Zr Alloy

**DOI:** 10.3390/ma11040570

**Published:** 2018-04-07

**Authors:** Aleksander Kowalski, Wojciech Ozgowicz, Wojciech Jurczak, Adam Grajcar, Sonia Boczkal, Janusz Żelechowski

**Affiliations:** 1 Institute of Non-Ferrous Metals, 5 Sowińskiego Street, 44-100 Gliwice, Poland; 2 Silesian University of Technology, Institute of Engineering Materials and Biomaterials, 18A Konarskiego Street, 44-100 Gliwice, Poland; wojciech.ozgowicz@tlen.pl (W.O.); adam.grajcar@polsl.pl (A.G.); 3 Polish Naval Academy, Faculty of Mechanical and Electrical Engineering, 69 Śmidowicza Street, 81-127 Gdynia, Poland; w.jurczak@amw.gdynia.pl; 4 Institute of Non-Ferrous Metals, Light Metals Division, 19 Piłsudskiego Street, 32-050 Skawina, Poland; sboczkal@imn.skawina.pl (S.B.); jzelechowski@imn.skawina.pl (J.Ż)

**Keywords:** aluminium alloy, 7003 alloy, corrosion resistance, thermomechanical treatment, TEM, X-ray diffraction

## Abstract

The paper presents results of the investigations on the effect of low-temperature thermomechanical treatment (LTTT) on the microstructure of AlZn6Mg0.8Zr alloy (7000 series) and its mechanical properties as well as electrochemical and stress corrosion resistance. For comparison of the LTTT effect, the alloy was subjected to conventional precipitation hardening. Comparative studies were conducted in the fields of metallographic examinations and static tensile tests. It was found that mechanical properties after the LTTT were better in comparison to after conventional heat treatment (CHT). The tested alloy after low-temperature thermomechanical treatment with increasing plastic deformation shows decreased electrochemical corrosion resistance during potentiodynamic tests. The alloy after low-temperature thermomechanical treatment with deformation degree in the range of 10 to 30% is characterized by a high resistance to stress corrosion specified by the level of P_SCC_ indices.

## 1. Introduction

The effect of microstructure and morphology of intermetallic phases on mechanical properties and corrosion resistance of Al–Zn–Mg wrought aluminium alloys is important due to the required properties of final products. The proper design of structural elements exposed to the aggressive impact of chloride ion medium requires comprehensive understanding of the relationships between microstructure, microsegregation of alloying elements, type and morphology of intermetallic precipitations, and the heat or thermomechanical treatment performed [1,2,3,4,5]. Refinement of microstructure and the size of precipitations, ensuring optimal strain hardening and precipitation strengthening of the 7000 series aluminium alloys as a result of thermomechanical processing, is an indispensable condition for technological development in the field of newly designed light constructions of machines and devices in many industrial fields [6,7,8,9].

Integration of heat treatment and plastic deformation operations of aluminium alloys significantly improves their impact efficiency on the microstructure, especially in the case of conventional techniques including cold rolling, solution heat treatment, and ageing. Advantageous effects of improved mechanical properties and corrosion resistance of Al–Zn–Mg alloys are obtained as a result of synergetic interactions of strain hardening and precipitation hardening [10,11,12,13]. Cold deformation of supersaturated solid solution reduces its thermodynamic stability and accelerates ageing effects. However, the influence of strain hardening on the ageing process is complicated, because it depends on conditions of supersaturation, deformation degree and ageing temperature, alloy type, and the type of precipitations occurring during ageing [14,15,16,17].

Nowadays, Al–Zn–Mg–Cu alloys and their various modifications with Zr, Sc, and Ti microadditions are most interesting among the high-strength aluminium alloys of the 7000 series. Deng et al. [11] investigated the possibility of counteracting recrystallization in the 7085 alloy by applying high-temperature thermomechanical treatment with subsequent precipitation hardening. In turn, Lee et al. [12] considered obtaining the fine-grained microstructure of the 7075 alloy using twin roll casting and thermomechanical treatment. Another modification of technological process of the 7075 alloy, which consisted of double thermomechanical processing leading to refinement of the microstructure, was proposed by El-Baradie et al. [13]. Zuo et al. [15,16,17] used two-stage hot rolling during thermomechanical treatment of the 7055 alloy for realization of the same purpose. In turn, Huo et al. [18,19] carried out continuous rolling during cooling from a hot rolling temperature for the 7075 alloy.

Adequate resistance in aggressive corrosive media is an important criterion in selecting Al–Zn–Mg alloys for responsible construction elements in various industries. Chemical composition becomes substantial in this aspect—primarily the total content of Zn and Mg, the amount of Cu, and microadditions of Zr, Sc, or Ti [20,21,22]. Moreover, microstructure development, ensuring high stress corrosion resistance while maintaining high mechanical properties, is also not to be neglected [23,24]. Wang et al. [25] investigated the stress corrosion resistance of an Al–Zn–Mg alloy with a small content of additions, subjected to two-stage precipitation hardening. Successively, comprehensive considerations on the effect of variant heat treatment of Al–Zn–Mg–Cu (7085) alloy on microstructure and mechanical properties, and, in particular, resistance to intercrystalline and stress corrosion, were the subject of research led by Peng et al. [26]. The effect of precipitation hardening parameters on microstructure of an Al–Zn–Mg–Sc–Zr alloy, which in turn determines stress corrosion resistance, was undertaken by Huang et al. [27]. Sun et al. [28] conducted research on the dependence of grain boundary structure and stress corrosion of an Al–Zn–Mg alloy without Cu and microadditions.

Available literature data mainly relate to Al–Zn–Mg alloys containing Cu. Difficulties in obtaining high-quality welded joints limit their application. Aluminium alloys of this series, with limited amounts of Cu, are superior in this aspect. There are, however, relatively few scientific studies on these alloys. The number of available publications dealing with the matter of thermomechanical treatment of the 7000 series alloys, and, in particular, its low-temperature variant with Zr microaddition, is also not high. Therefore, the development of knowledge, enabling optimal use of available methods of plastic working and heat treatment as well as their synergetic effect on improvement of functional properties of 7000 series aluminium alloys without the addition of Cu, is desirable. This is why studies of AlZn6Mg0.8Zr alloy were aimed at determining the impact of different reductions during low-temperature thermomechanical treatment on its microstructure, mechanical properties, and resistance to electrochemical and stress corrosion.

## 2. Materials and Methods

### 2.1. Materials and Heat Treatment

**Figure 1:**
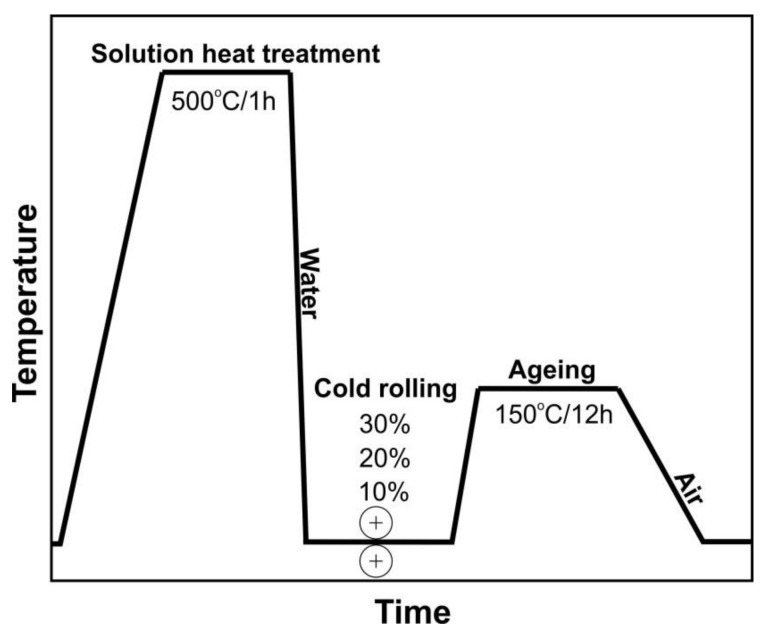
A schematic diagram of the low-temperature thermomechanical treatment (LTTT) of the AlZn6Mg0.8Zr alloy.

The material used was a metal sheet, cold-rolled from Al–Zn–Mg (7000 series) commercial aluminium alloy with the following chemical composition: 6.13 Zn, 0.74 Mg, 0.30 Mn, 0.20 Fe, 0.17 Cr, 0.12 Si, 0.08 Zr, 0.04 Cu, and the remainder Al. The investigated alloy was subjected to the following low-temperature thermomechanical treatment: supersaturation in water from the temperature of 500 °C after a holding time of 1 h; cold-rolled with a reduction of 10%, 20%, and 30%; strain-aged for 12 h at a temperature of 150 °C; then cooled down in the open air (Figure 1). For comparison purposes, the tested alloy was subjected to the following precipitation hardening: solution heat treatment (supersaturation) in water from the temperature of 500 °C after a holding time of 1 h; quench-aged for 12 h at a temperature of 150 °C; then cooled down in the open air.

### 2.2. Tensile Test

Static tensile tests of the AlZn6Mg0.8Zr alloy were carried out at room temperature after low-temperature thermomechanical treatment and conventional precipitation hardening using an INSTRON 4505 universal testing machine at a traverse speed rate of 2 mm/min, according to the standard [29]. For examinations of mechanical properties, round samples (three for each state) with the following dimensions of the measuring part were used: Ø 10 mm and l_o_ = 60 mm. The presented results are an arithmetic average of three measurements.

### 2.3. Transmission Electron Microscopy

Transmission electron microscopy (TEM) tests were carried out using the thin foil technique. Discs of Ø 3 mm diameter, cut from the AlZn6Mg0.8Zr alloy after low-temperature thermomechanical treatment (LTTT) and conventional heat treatment (CHT), were ground on abrasive paper to a thickness of about 0.3 mm, and then electrolytically polished at temperature −20 °C at voltage 16.5 V in Struers A2 reagent using a TenuPol-5 device. The microstructure of the thin foils was observed using a TECNAI G2 (FEI) transmission electron microscope, at accelerating voltage of 200 kV and magnification up to 120000×. The phase identification procedure, based on electron diffraction, was performed with the aid of the ELDYF computer program [30].

### 2.4. X-ray Diffraction Examinations

X-ray examinations were performed in the delivery state and after conventional heat treatment and LTTT operations. For this purpose, the D8 Advance (Bruker, Karlsruhe, Germany) X-ray diffractometer was used. The XRD pattern was obtained with the Bragg–Brentano method using X-ray radiation with energy of approximately 8.041 keV, which corresponds to with a wavelength of approximately 1.54184 Å. X-ray qualitative analysis was carried out in the range of θ angles from 10° to 40°. In order to obtain high-quality diffraction patterns of high accuracy and precision, the time of measurement cycle (step) was set to 100 s, which increases the recording time of one diffraction line ranging from a few to several hours at the step equal to 0.045° θ.

### 2.5. Electrochemical and Stress Corrosion Examinations

Electrochemical corrosion resistance tests were performed using the potentiodynamic method, in accordance with recommendations of the standard [31]. Corrosion resistance was analyzed on the basis of registered potentiodynamic curves of a logarithm dependence of current density versus potential value. Measurements were carried out in the 3.5% NaCl medium (pH = 7). The examinations were performed on three specimens for each state after LTTT and the arithmetic average of measurements is presented.

**Figure 2:**
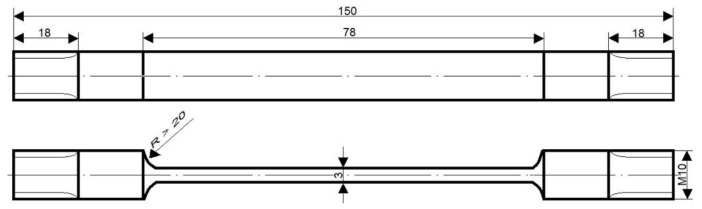
Shape and dimensions of stress corrosion test specimens.

Stress corrosion resistance tests were executed at the PNA Gdynia stand at a constant tensile load of σ_o_ = (0.8)R_p0.2_ (yield strength) in the medium of 3.5% NaCl aqueous solution. The corrosion test temperature was within the range of room temperature, and the NaCl aqueous solution was changed every two days. The shape of specimens is presented in Figure 2. The exposure time was equal to 1512 h, and three specimens for each state were used. The resistance of the tested alloy to stress corrosion was determined by comparing mechanical properties obtained in a static tensile test before and after corrosion exposure, using the following Formula (1):

(1) $$P_{\mathrm{SCC}}=\left(1-\frac{P_{\mathrm{NaCl}}}{P_{\mathrm{air}}}\right)\cdot 100\%$$

where

P_SCC_—stress corrosion susceptibility index for individual material properties;
P_NaCl_—material property measured in corroding medium;
P_air_—material property measured in air.

## 3. Results and Discussion

### 3.1. Mechanical Properties and Microstructure

**Figure 3:**
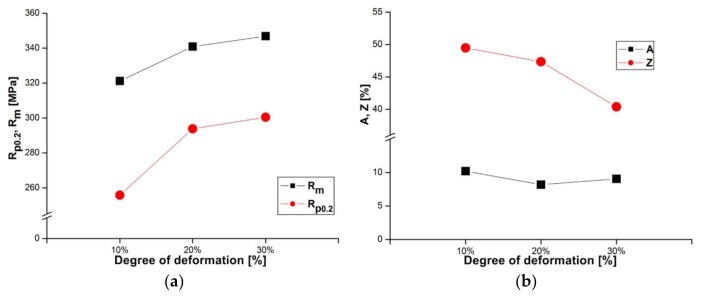
Influence of deformation degree on (**a**) strength and (**b**) plastic properties of the investigated alloy after LTTT.

The AlZn6Mg0.8Zr alloy subjected to LTTT reveals a yield point from approximately 256 MPa to about 300 MPa and tensile strength from approximately 321 MPa to 347 MPa, depending on the degree of cold deformation after solution heat treatment in a range from 10 to 30% (Figure 3a). The obtained values of tensile strength are lower (approximately 250 MPa) in comparison with an alloy containing 8.38% Zn, 2.07% Mg, 2.31% Cu, and 0.13% Zr subjected to high-temperature thermomechanical treatment (HTTT) [15,16,17]. However, the strength difference decreases with the reduction of Cu and Zr contents. For example, an alloy containing 7.81% Zn, 1.62% Mg, 1.81% Cu, and 0.13% Zr after HTTT is characterized by 160 MPa higher tensile strength [11], while an alloy with a copper content limited to 0.1% Cu and 0.13% Zr subjected to more complex heat treatment shows a 30 MPa increase in tensile strength [8]. It should be noted that these values depend significantly on processing conditions. The value of the R_p0.2_/R_m_ ratio varies along with the deformation degree from 0.8 to 0.87. Elongation of the examined alloy takes similar values, whereas reduction of area decreases by approximately 9% for a given deformation range (Figure 3b). Obtained values of mechanical properties after thermomechanical treatment are higher when compared to the values obtained after CHT (Table 1). A significant rise in yield point has been noted. Thermomechanical treatment causes a slight decrease of elongation. Nevertheless, in comparison to the alloy subjected to the CHT, the tested alloy subjected to the LTTT is characterized by higher strength and reduction of area in the entire range of deformation.

The R_p0.2_/R_m_ ratio should also be taken into consideration. The AlZn6Mg0.8Zr alloy subjected to LTTT is characterized by a higher R_p0.2_/R_m_ ratio (about 0.87), in comparison to the CHT carried out (about 0.74) under comparable processing conditions. Generally, higher values of this ratio are observed for aluminium alloys with a smaller fraction of recrystallized microstructure, higher strengthening from the substructure, and lower elongation [11], which corresponds well with obtained results.

**Figure 4:**
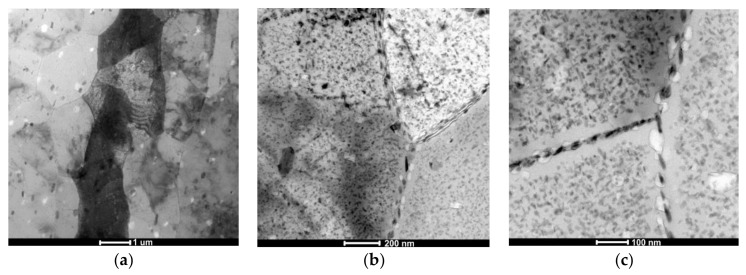
The AlZn6Mg0.8Zr alloy after CHT: (**a**) substructure; (**b**) precipitations inside grains and along grains’ boundaries; (**c**) precipitation-free zones (PFZ) near to grains’ boundaries with continuously distributed precipitates.

**Figure 5:**
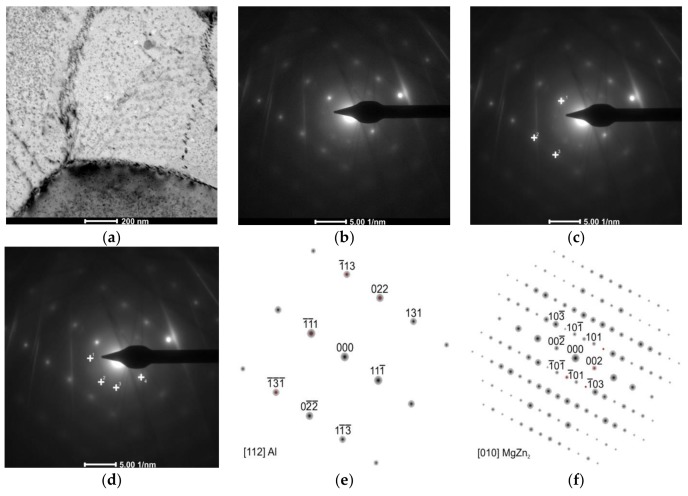
The AlZn6Mg0.8Zr alloy after CHT: (**a**) substructure of investigated alloy with stochastic distribution of precipitations inside subgrains and along their boundaries; (**b**) electron diffraction; (**c**) marked reflections of α solution matrix; (**d**) marked reflections of MgZn_2_ particle; (**e**) solution of α phase matrix diffraction and (**f**) solution of MgZn_2_ phase diffraction.

Observations of thin foils using transmission electron microscopy (TEM) revealed after the CHT the substructure of the α solution matrix composed of subgrains ranging from 1 μm to about 2 μm, inside which fine, oval, and nonuniformly distributed precipitates were found (Figure 4a). The presence of grain boundaries with particles arranged along these boundaries (Figure 4b), often in a continuous manner (Figure 4c), was also revealed. These precipitates are also distributed uniformly inside grains or they are stochastic. It has also been found that there are precipitation-free zones (PFZ) in the vicinity of grain boundaries (Figure 4c). This microstructure is characteristic for Al–Zn–Mg alloys subjected to heat treatment [25,26,27]. Deschamps et al. [9] found that the critical factor shaping the microstructure of Al–Zn–Mg–Zr alloys, in addition to degradation and morphology of the Al_3_Zr phase, is also the volume fraction of GP zones, because they are privileged nucleation areas of the η’ phase. Electron diffractions revealed reflexes coming from the α solution matrix (Al) and MgZn_2_ phase (Figure 5).

**Figure 6:**
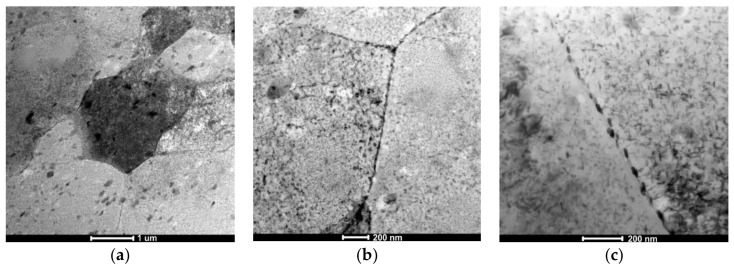
The AlZn6Mg0.8Zr alloy after LTTT with 10% deformation: (**a**) α solution matrix with nonuniform distribution of precipitates; (**b**) fine chain-arranged particles along grain boundaries; (**c**) PFZ along grain boundary with precipitates.

**Figure 7:**
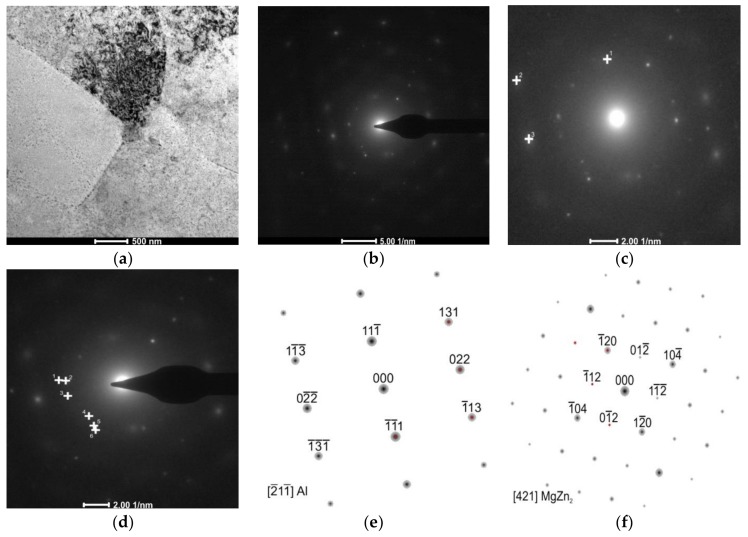
The AlZn6Mg0.8Zr alloy after LTTT with 10% deformation: (**a**) stochastically distributed precipitates in the substructure; (**b**) electron diffraction; (**c**) marked reflections of α solution matrix; (**d**) marked reflections of MgZn_2_; (**e**) solution of α phase matrix diffraction and (**f**) solution of MgZn_2_ phase diffraction.

The alloy subjected to the LTTT with 10% reduction is characterized by the microstructure composed of subgrains of the α solution with size in the range of 0.5–1 μm (Figure 6a). Fine, oval precipitates are usually distributed along grain boundaries (Figure 6b). They do not form continuous nets. Along these boundaries, a narrow precipitation-free zone can be observed—less distinct than in the case of the CHT (Figure 6c). Diffraction analysis of these particles allowed the identification of η–MgZn_2_ particles (Figure 7).

**Figure 8:**
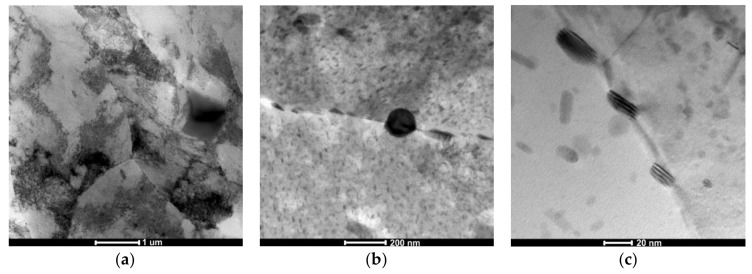
The AlZn6Mg0.8Zr alloy after LTTT with 30% deformation: **(a)** cellular dislocation structure; (**b**) chain-arranged system of precipitates along subgrain boundary; (**c**) morphology of MgZn_2_ particle.

**Figure 9:**
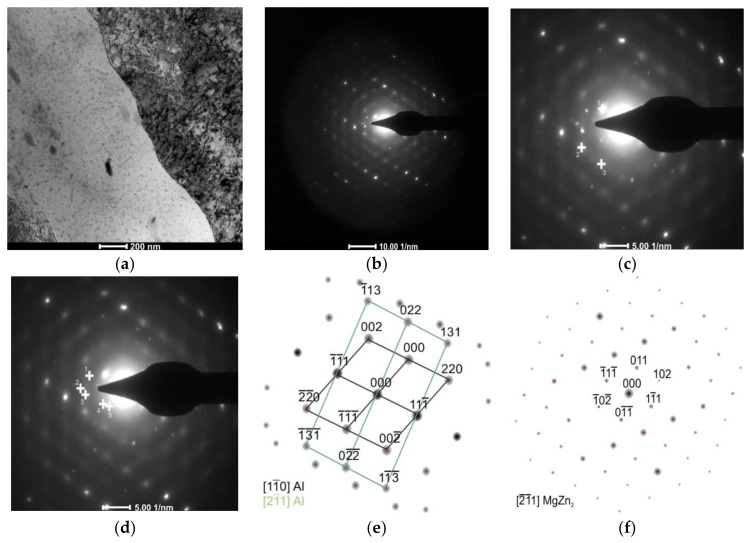
The AlZn6Mg0.8Zr alloy after LTTT with 30% deformation: (**a**) irregular subgrain boundaries; (**b**) electron diffraction; (**c**) marked reflections of α solution matrix; (**d**) marked reflections of MgZn_2_ precipitate; (**e**) solution of α phase matrix diffraction and (**f**) solution of MgZn_2_ phase diffraction.

Observations of thin foils of the alloy subjected to LTTT with 30% reduction revealed the presence of a cellular dislocation microstructure with a high density of dislocations (Figure 8a). This effect is caused by inhibition of the mobile dislocation movement by η phase precipitates and Al_3_Zr [15,18]. Numerous nonuniformly distributed precipitates were revealed in the matrix of the α phase. They are elongated, oval, and concentrated both inside grains and at their boundaries. It was found that particles are distributed discontinuously along grain and subgrain boundaries. Decay of precipitation-free zones in the vicinity of these boundaries was also observed (Figure 8b). Precipitates at the boundaries of grains reveal a characteristic arrangement of parallel lines on the surface with relatively regular spacing, which in the literature has been called the “striation effect”, characteristic for nanometric-sized precipitates [32]. This effect is metallographically similar to the patterns of dense stacking faults (Figure 8c). Based on electron diffraction, the presence of the MgZn_2_ phase was confirmed in the substructure of the alloy (Figure 9).

Microstructure observations of the alloy after the LTTT indicate a substantial role of plastic deformation, which influences formation of a substructure, primarily in the aspect of secondary phase precipitates. This is the result of the formation of a higher density of dislocations in the supersaturated α solution, which not only accelerate disintegration of this solution, but act as preferred nucleation areas of precipitation even before accelerated ageing during deformation, in the process of dynamic strain ageing and as an effect of hereditary impact—so-called static strain ageing—while soaking the alloy at the ageing temperature [15]. This is due to an increased diffusion rate in these processes and the presence of numerous nuclei in the deformed α solution, as well as faster coagulation of these phases. Similar results of the impact of plastic deformation on the microstructure of Al–Zn–Mg-type alloys have been presented in the literature [16,17,18,19]. The discussed changes in microstructure significantly affect obtained mechanical properties and, in particular, the dependencies between the level of strength and plastic properties [33,34]. On the one hand, attention is paid to the impact of the increased amount of precipitates formed in the LTTT process on the level of alloy strengthening. On the other hand, it influences a morphology, especially the degree of coagulation and distribution of formed particles. They affect a higher level of reduction in area after the LTTT compared with after conventional heat treatment.

### 3.2. Precipitation Behavior

**Figure 10:**
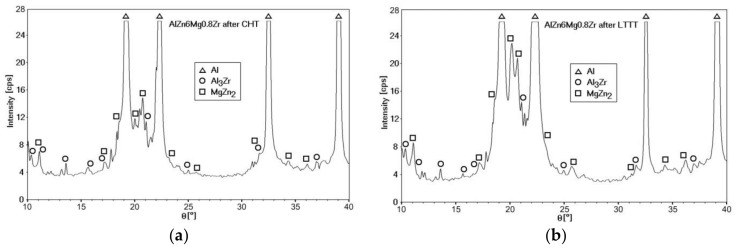
XRD patterns of the AlZn6Mg0.8Zr alloy after: (**a**) CHT (T_s_ = 500 °C; T_a_ = 150 °C; τ_a_ = 12h); (**b**) LTTT (T_s_ = 500 °C; 30%; T_a_ = 150 °C; τ_a_ = 12h).

Qualitative phase analysis of the AlZn6Mg0.8Zr alloy after the ageing stage in the CHT process revealed diffraction reflexes from the α matrix (Al) and Al_3_Zr and MgZn_2_ phases (Figure 10a). The same phases were identified in the alloy subjected to 30% cold deformation (Figure 10b). Moreover, the analysis of the intensity of recorded diffraction lines of the α solution matrix (Al), especially in case of the LTTT, indicates the lack of their compatibility with standard data.

The MgZn_2_ phase is most often identified by X-ray phase analysis [15,19,21,25]. Extending the time of the measurement step to 100s allowed the obtaining of more accurate diffraction patterns, which enabled the identification of Al_3_Zr dispersoids. The presence of fine-dispersive Al_3_Zr phases in alloys of the 7000 series, which are formed already during cooling of the alloy after solution heat treatment, is important because they can become privileged areas of heterogeneous nucleation of the strengthening η–MgZn_2_ phase [7]. The occurrence of Al_3_Zr particles in combination with the high dislocation density, in the case of the LTTT processing, causes formation of more nucleation sites for the η phase and, thus, a higher strength of alloys containing Zr [9,11].

### 3.3. Corrosion Resistance

**Figure 11:**
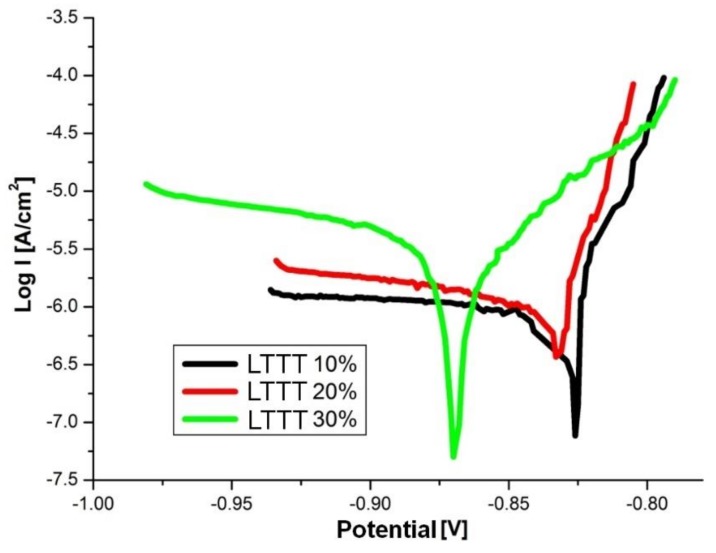
Polarization curves of the alloy after the LTTT with plastic deformation of 10%, 20%, and 30%.

The AlZn6Mg0.8Zr alloy subjected to the LTTT with 10% and 20% reductions has a comparable corrosion potential (approximately −830 mV) (Figure 11). An increase in degree of deformation to 30% causes a decrease in E_cor_ by approximately 35 mV toward more active values. The value of the I_cor_ index increases and R_p_ decreases along with the increase of the deformation (Table 2). It was found that increase of the deformation amount leads to a decrease of electrochemical corrosion resistance. This is due to the formation of larger fraction of precipitates. They interact with the matrix and undergo anodic stripping, leading to the deterioration of electrochemical pitting corrosion factors [21,22,28].

The alloy subjected to the LTTT is characterized by a slight decrease in mechanical properties in a static tensile test after loading the samples under stress corrosion conditions (Table 3). It was found that the decrease of mechanical factors after stress and corrosion exposures of samples is reduced when the degree of deformation rises. High values of stress corrosion susceptibility indices of the tested alloy (P_SCC_) allow us to state that the AlZn6Mg0.8Zr alloy, subjected to the LTTT, is not very susceptible to this type of corrosion (Table 4). Similar conclusions for an Al–Zn–Mg–Zr alloy containing a small amount of Cu, subjected to various conventional heat treatments, were obtained by Xiao et al. [8]. In their case, the decrease in mechanical properties was more pronounced. It was also observed that P_SCC_ indices, for certain strength properties, are smaller than plastic properties indices (Z_SCC_ in particular). This indicates that strength properties are less susceptible to the effects of stress corrosion conditions. A decrease in all P_SCC_ indexes was also noted along with an increase in the deformation degree, which reflects improved stress corrosion resistance of the alloy along with its strengthening.

Cold deformation after solution heat treatment results in formation of a dislocation network, providing privileged areas for heterogeneous nucleation of the η phase. Moreover, new diffusion paths are created, which affect the precipitation sequence of the strengthening phases. As a consequence, the amount of η phase formed in the deformed alloy is larger than the portion of η’ phase, which positively influences the resistance under stress corrosion conditions [24]. Moreover, ageing activated diffusion of alloying elements from inside to grain boundaries of cold-deformed alloys. This results in growth and coagulation of precipitates at the boundaries and their distribution in large intervals between them. The precipitation-free zone is also reduced [25,26,27,35].

## 4. Conclusions

Studies of mechanical properties and corrosion resistance of the 7003 series Al–Zn–Mg-type alloy and metallographic analyses lead to the following conclusions:

- Low-temperature thermomechanical treatment with 30% reduction after solution heat treatment in water from the temperature of 500 °C and with ageing at the temperature of 150 °C ensures higher mechanical properties of the alloy in comparison to CHT.
- AlZn6Mg0.8Zr alloy reveals a microstructure consisting of the α solution matrix and fine-dispersive particles of morphologically differentiated intermetallic phases of the Al_3_Zr and η–MgZn_2_ types. Increase of cold deformation results in obtaining a smaller precipitation-free zone after ageing and formation of a greater portion of strengthening phases in the vicinity of grain boundaries. This affects the increase in the distance between precipitates located at grain boundaries.
- Electrochemical corrosion resistance of the AlZn6Mg0.8Zr alloy in the 3.5% NaCl medium decreases along with increasing plastic deformation degree, whereas the opposite behavior occurs in the case of stress corrosion resistance, which is improved. This is related to complex microstructural phenomena, which must be studied in more detail.

## Figures and Tables

**Table 1:** Mechanical properties of the investigated alloy after the LTTT and conventional heat treatment (CHT).

Treatment Parameters				Mechanical Properties			
LTTT							
Temperature of Solution Heat Treatment (°C)	Temperature of Ageing (°C)	Time of Ageing (h)	Degree of Deformation (%)	$\bar{R_{p0.2}}$ (MPa)	$\bar{R_{m}}\;$(MPa)	$\bar{A}$(%)	$\bar{Z}$(%)
500	150	12	10%	256 ± 4	321 ± 2	10.2 ± 0.4	50 ± 5
			20%	294 ± 2	341 ± 2	8.2 ± 1.1	47 ± 5
			30%	301 ± 2	347 ± 2	9.1 ± 0.9	40 ± 4
**CHT**							
500	150	12	–––	230 ± 3	310 ± 2	16.8 ± 0.5	34 ± 4

R_m_—tensile strength; A—total elongation; Z—reduction in area.

**Table 2:** Electrochemical parameters obtained from cyclic polarization measurements.

Degree of Deformation (%)	E_cor_ (mV)	I_cor_ (μA/cm^2^)	R_p_ (kΩ)
10	−830 ± 1	0.30 ± 0.2	17.4 ± 0.5
20	−833 ± 3	1.32 ± 0.3	6.2 ± 0.2
30	−867 ± 4	11.17 ± 0.7	5.4 ± 0.9

**Table 3:** Mechanical properties of AlZnMg0.8Zr alloy, subjected to LTTT, obtained after and before stress corrosion tests.

Degree of Deformation (%)	$\bar{R_{p0.2}}$ (MPa)	$\bar{R_{m}}$ (MPa)	$\bar{A}$ (%)	$\bar{Z}$ (%)
**Before Corrosion**				
10	256 ± 4	321 ± 2	10.2 ± 0.4	50 ± 5
20	294 ± 2	341 ± 2	8.2 ± 1.1	47 ± 5
30	301 ± 2	347 ± 2	9.1 ± 0.9	40 ± 4
**After Corrosion**				
10	249 ± 2	316 ± 2	9.8 ± 1.3	46 ± 3
20	289 ± 3	337 ± 3	8.0 ± 0.7	45 ± 5
30	300 ± 4	346 ± 4	8.9 ± 0.5	39 ± 5

**Table 4:** P_SCC_ indices for the investigated alloy after LTTT.

Degree of Deformation (%)	P_SCC_ Index (%)			
	R_p0.2SCC_	R_mSCC_	A_SCC_	Z_SCC_
10	2.7	1.7	3.9	7.9
20	1.7	1.1	2.4	5.5
30	0.2	0.2	2.2	4.7
